# Death Patterns during the 1918 Influenza Pandemic in Chile

**DOI:** 10.3201/eid2011.130632

**Published:** 2014-11

**Authors:** Gerardo Chowell, Lone Simonsen, Jose Flores, Mark A. Miller, Cécile Viboud

**Affiliations:** Arizona State University, Tempe, Arizona, USA (G. Chowell);; National Institutes of Health, Bethesda, Maryland, USA (G. Chowell, M.A. Miller, C. Viboud);; George Washington University, Washington, DC, USA (L. Simonsen);; University of South Dakota, Vermillion, South Dakota, USA (J. Flores);; Universidad de Chile, Santiago, Chile (J. Flores)

**Keywords:** 1918 influenza pandemic, Chile, Concepción, age-specific mortality, geography, population density, latitudinal gradient, viruses, influenza

## Abstract

Scarce information about the epidemiology of historical influenza pandemics in South America prevents complete understanding of pandemic patterns throughout the continent and across different climatic zones. To fill gaps with regard to spatiotemporal patterns of deaths associated with the 1918 influenza pandemic in Chile, we reviewed archival records. We found evidence that multiple pandemic waves at various times of the year and of varying intensities occurred during 1918–1921 and that influenza-related excess deaths peaked during July–August 1919. Pandemic-associated mortality rates were elevated for all age groups, including for adults >50 years of age; elevation from baseline was highest for young adults. Overall, the rate of excess deaths from the pandemic was estimated at 0.94% in Chile, similar to rates reported elsewhere in Latin America, but rates varied ≈10-fold across provinces. Patterns of death during the pandemic were affected by variation in host-specific susceptibility, population density, baseline death rate, and climate.

## Abstract 2

Death rates varied by region, but not age group, and peaked during July–August 1919.

The 1918 Influenza Pandemic in Chile

 The 1918 influenza A(H1N1) pandemic was one of the most devastating epidemic events in recent history; an estimated ≈1% of the global population (20–50 million persons) died (*1*), including >14 million in India alone (*2*). Our understanding of the epidemiologic patterns of this pandemic has improved over the past decade as a result of intensive efforts to locate, digitize, and analyze archival disease records (*3*). In particular, studies focusing on the United States (*4*,*5*), Mexico (*6*), Colombia (*7*), Brazil (*8*), and Peru (*9*) have shed light on geographic variation in the patterns of timing, intensity, and patient age during successive pandemic waves across the Americas. Yet there have been no reports from temperate locales of South America; this gap prevents a complete understanding of the epidemiology of the pandemic throughout the continent and across different climatic zones.

A consistent finding across reports from North America, Europe, Latin America, and Asia (*4*–*7*,*9*–*12*) is the disproportionate increase in mortality rates among young adults during the pandemic period compared with prepandemic years. Further reports from the United States and Europe have shown that influenza-related deaths among seniors (>50 years of age) were significantly reduced during the lethal 1918–19 pandemic wave relative to baseline periods. In contrast, this protective effect for seniors did not apply to Mexico, Colombia, and remote island populations, probably because of differences in prior immunity between regions (*6*,*13*). In addition, large geographic variations in mortality rates from the pandemic have been documented (*14*–*16*).

Although recent studies have highlighted latitudinal and climatic variations in contemporary influenza epidemics and pandemics (*17*–*20*), little is known about the role of climate in the severity or timing of the 1918 pandemic. Chile is a unique country; it spans an extensive latitudinal gradient, has a variety of climatic zones, and has preserved historical records dating back to the 1918 pandemic period. We characterized geographic variation in pandemic influenza–related death patterns in Chile by using national and regional death statistics combined with a unique dataset of individual-level death certificates from Concepción, a large city in southern Chile. We discuss our findings in relation to those reported elsewhere in the Americas and globally.

## Material and Methods

### Geographic Setting

**Figure 1:**
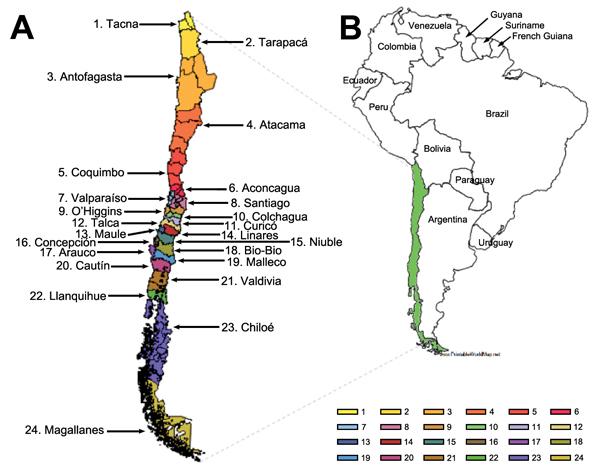
The 24 contiguous administrative provinces in Chile in 1918, (A) and location of Chile in South America.

Chile covers a long and narrow strip between the Andes mountains (to the east) and the Pacific Ocean (to the west), located at latitude 17°S–56°S and longitude 66°W–76°W. The climatic zones of Chile include dry and desert areas in the north, Mediterranean regions in the center, and rainy temperate areas in the south. According to census information, the 1917 population of Chile was 3.9 million (*21*), and Chile was divided into 24 contiguous administrative provinces (Figure 1). In the period surrounding the 1918 pandemic, hygienic conditions were poor and the infant mortality rate was very high (rate among children <1 year of age was 25%–30%) (*21*).

### Data Sources

#### Vital Statistics, Chile, Anuarios Estadísticos, 1915–1921

To explore the spatiotemporal patterns of pandemic-related deaths in Chile, we compiled monthly all-cause death statistics for 1915–1921 for the 24 administrative provinces from official publications and a variety of sources (*21*). We obtained province-level population size estimates (1917 census), population density estimates (1920), and infant (<1 year of age) mortality rates (*21*) (Table 1). To explore pandemic timing and effect along a latitudinal gradient, we compiled geographic coordinates for province-level population centers.

**Table 1:** Estimates of absolute excess mortality rates attributable to pandemic influenza across 24 provinces of Chile, 1918–1921*

Province†	Pandemic period, no. excess deaths/10,000 population				Cumulative absolute excess mortality rate
	Jul 1918–Mar 1919	Jun 1919–Mar 1920	Nov 1920–Mar 1921	Jun 1921–Dec 1921	
Tacna	8.8	64.8	0.0	0.0	73.6
Tarapacá	14.3	0.0	10.5	0.0	24.8
Antofagasta	0.0	0.0	0.0	0.0	0.0
Atacama	27.0	27.5	0.0	42.3	96.8
Coquimbo	0.0	59.2	0.0	38.3	97.5
Aconcagua	0.0	44.6	0.0	0.0	44.6
Valparaiso	20.6	36.5	0.0	0.0	57.1
Santiago	48.0	57.9	0.0	99.7	205.6
O’ Higgins	15.1	37.4	8.0	0.0	60.5
Colchagua	12.7	57.6	0.0	0.0	70.3
Curicó	9.7	44.5	0.0	0.0	54.2
Talca	0.0	53.0	0.0	0.0	53
Maule	0.0	56.8	0.0	0.0	56.8
Linares‡	0.0	25.6	0.0	0.0	25.6
Niuble	7.2	70.0	0.0	0.0	77.2
Concepción	0.0	57.0	0.0	0.0	57
Arauco	0.0	41.0	8.5	0.0	49.5
Bio-Bio	0.0	125.2	0.0	0.0	125.2
Malleco	0.0	76.6	0.0	0.0	76.6
Cautin	6.4	52.6	0.0	0.0	59
Valdivia	0.0	56.7	11.2	30.0	97.9
Llanquihue	7.6	80.2	0.0	36.3	124.1
Chiloé	0.0	84.1	0.0	62.3	146.4
Magallanes	21.8	18.9	0.0	46.0	86.7
Total Chile	13.6	54.4	1.4	24.5	93.9

*Excess mortality estimates were based on a seasonal regression model applied to monthly deaths.  †Provinces are sorted in geographic order from northern to southern Chile. ‡Model baseline to estimate excess deaths excluded the high-death months of Sep 1917–Mar 1918.

#### Historical Death Certificates in Concepción, Chile, 1915–1920

Given the lack of age detail in official vital statistics records, we used a different data source to document the age patterns of persons who died during the pandemic. Concepción is the third largest city in the country and has kept well-preserved individual death certificates. All-cause death records comprising 18,805 individual death certificates corresponding to January 1915–December 1920 were manually retrieved from the civil registry of the city. For each record, we tabulated the person’s age at death, cause of death, and exact date of death. Most death certificates listed a unique cause of death. We then created a weekly time series for deaths from all causes and respiratory illnesses, stratified into 4 age groups (<20, 20–29, 30–49, and >50 years). Deaths ascribed to influenza, pneumonia, bronchopneumonia, or bronchitis were classified as respiratory. Information about age or cause of death was missing on <1% of all records. We obtained age-specific population data for Concepción from the Instituto Nacional de Estadísticas and used estimates from 1920 to derive age-specific mortality rates for the study period (*22*). The total population of the city was 82,662 in 1920, and it increased ≈1% annually over the study period (*22*)

#### Local Newspapers, 1918–1919

We gathered anecdotal information on the temporal course and severity of the pandemic during 1918–1919 by examining the most popular daily newspapers published in the capital city of Santiago (El Mercurio) and in Concepción (El Sur). We identified 49 issues of El Mercurio and 45 issues of El Sur that contained articles or commentaries relating to pandemic influenza during this period.

### Statistical Analyses

To quantify the mortality rate associated with the influenza pandemic by province and age group during 1918–1921, we estimated excess deaths as the number of deaths occurring above a seasonal baseline of expected deaths in the absence of pandemic influenza activity (*23*). To estimate baseline deaths in the absence of influenza activity, we fitted a cyclical regression model to monthly mortality data for the prepandemic period 1915–1917 and included temporal trends and harmonic terms for seasonality (*5*,*24*–*26*). For the province of Linares, we excluded observations for months with atypically high numbers of deaths during September 1917–March 1918 from the baseline model. Periods with statistically significant elevations in deaths over the model baseline were interpreted as periods of pandemic-related excess deaths.

Pandemic periods for each province were defined as the months when all-cause deaths exceeded the upper 95% confidence limit of the baseline model. We then summed the excess deaths above the baseline model during each pandemic period identified during 1918–1921 to estimate the absolute mortality burden of the pandemic.

We also calculated the relative risk (RR) for pandemic-associated death, defined as the ratio of excess deaths during the pandemic periods to the expected baseline number of deaths for these periods. The RR measure facilitates comparison between countries, regions, and patient age groups with different background risks for death (*25*,*27*).

### Geographic Patterns

We analyzed geographic variations in pandemic timing and severity across 24 provinces spanning the entire latitudinal gradient of Chile (Figure 1). For each geographic area, we recorded the timing of the pandemic all-cause death peak, defined as the month with maximal elevation of deaths during 1918–1921. We also used univariate analyses and multivariate stepwise regression models to explore the association between the variables of province-level estimates of peak timing, excess mortality rate, and RR for death and the variables of latitude, population size, population density, and infant mortality rates. 

### Spatial Autocorrelation

We quantified the extent of spatial autocorrelation in estimates of excess deaths and RR in the 24 provinces by using the Moran I statistic and a nearest-neighbor spatial mixing matrix (*28*) via randomization tests (*29*). This analysis evaluates whether pandemic mortality rates in contiguous provinces are more similar than those obtained for any pair of provinces selected at random.

## Results

### Newspaper Information

The first newspaper report of the pandemic in Santiago, Chile, appeared in the El Mercurio newspaper on October 16, 1918. Although during the early stages of the pandemic, the etiology was suspected to be typhus exanthematicus, the clinical characteristics readily favored a respiratory disease, reminiscent of influenza illnesses that had affected other areas of the world several months earlier. By October 29, 1918, some hospitals in Santiago City had reached maximum capacity. In Concepción, the first reports referring to the pandemic were published on October 24, 1918, in the local newspaper, El Sur.

### Geographic Patterns, Pandemic Waves, and Excess Deaths

**Figure 2:**
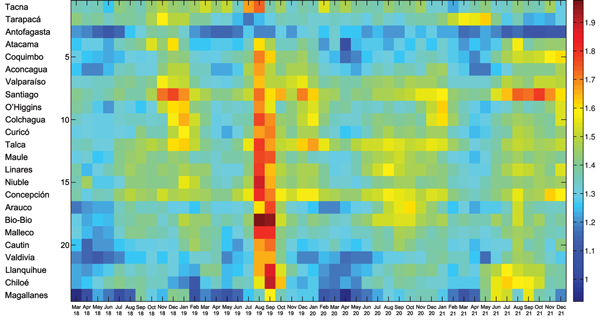
Temporal evolution (March 1918–December 1921) of all-cause mortality rates during the 1918 influenza pandemic across 24 provinces of Chile, sorted in geographic order from northern to southern Chile. For visualization purposes, the time series are log transformed.

According to province-level all-cause monthly mortality rates, the pandemic virus spread heterogeneously in multiple waves across Chile (Figure 2). The first pandemic wave spanned from October 1918 through February 1919, peaked in January 1919, and was relatively mild. The mean excess mortality rate was 13.6 deaths per 10,000 population across provinces. Latitude and population size explained 33% of the variability in the timing of peak pandemic activity from northern to southern Chile; deaths peaked earlier in the northern provinces (p = 0.02) (Figure 2).

**Figure 3:**
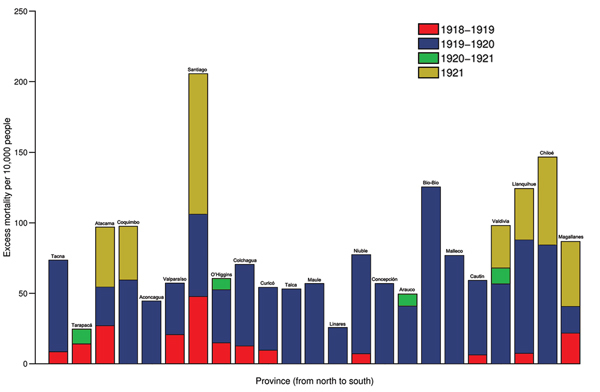
Excess deaths per 10,000 population across 24 provinces of Chile according to pandemic periods (July 1918–March 1919, June 1919–March 1920, November 1920–March 1921, and June 1921–December 1921) in geographic order from northern to southern Chile. Excess deaths are above the upper limit of the baseline mortality curve calibrated by using all-cause monthly deaths before the 1918 influenza pandemic.

**Figure 4:**
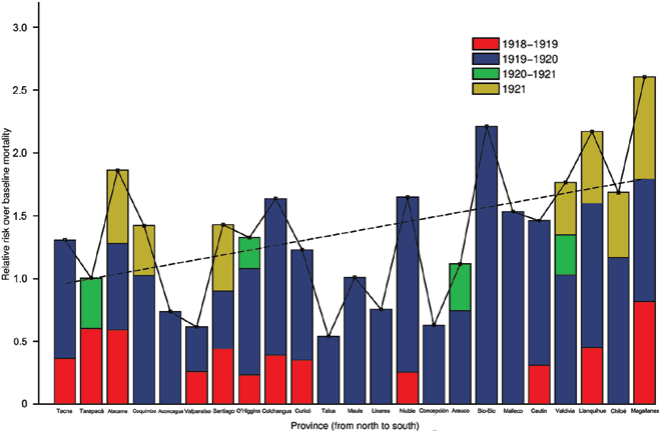
Relative risk for death over the baseline risk across provinces of Chile according to pandemic periods (July 1918–March 1919, June 1919–March 1920, and June 1921–December 1921). The relative risk for death is based on the ratio of excess deaths to baseline deaths, facilitating comparisons across provinces with different background risks for death. Solid line shows how relative risk varies with latitude by connecting observations for individual provinces; dashed line is a fitted linear trend.

The highest absolute excess mortality rates in this period occurred in Santiago (Table 1; Figure 3). Baseline mortality rates and population density explained 89% of the variability in excess mortality rates across provinces during the 1918–1919 wave (p<0.0001). The province in which RR for excess pandemic deaths was highest was the southernmost province of Magallanes (RR 81.5%) (Figure 4).

Chile experienced the brunt of pandemic deaths during the second wave in the winter–spring of July 1919–February 1920; deaths peaked in August 1919. The average excess mortality rate was 54.4 deaths per 10,000 population across provinces (Figure 3); all provinces except the northern provinces of Tarapacá and Antofagasta experienced substantial excess mortality rates during this period (Table 1). Baseline mortality rates and population density explained 29.9% of the variability in excess mortality rates (p = 0.03). The RR for death ranged from 0 (Tarapacá) to 221% (Bio-Bio in southern Chile); the RR trend increased significantly from northern to southern Chile, even after population size was controlled for (Spearman ρ = 0.56, p = 0.007) (Figure 4).

During spring–summer 1920–1921, a third minor pandemic wave affected 4 provinces in northern (n = 1), central (n = 1), and southern Chile (n = 2) (Table 1). This mild wave was followed by a fourth wave of pandemic activity during June–December 1921, which generated excess deaths in 7 provinces (Table 1, Figure 3). The effect of this fourth pandemic wave was highest in Santiago; excess mortality rate was 99.7 deaths per 10,000 population, corresponding to a 42.8% elevation over baseline (Figures 3, 4).

The cumulative excess mortality rate during the 1918–1921 study period was estimated at 93.9 deaths per 10,000 population (RR 129%), representing 34,978 excess deaths nationally. Cumulative excess pandemic death rates varied by ≈10-fold across provinces, from 24.8 deaths per 10,000 population in Tarapacá to 205.6 per 10,000 in Santiago, the hardest hit province. Stepwise multivariate regression identified baseline mortality rates and population density as significant and positively associated predictors of cumulative excess mortality rates across provinces (R^2^ = 71.4%, p<0.0001). There was no association between cumulative excess mortality rates and latitude (Spearman ρ = 0.32, p = 0.14).

In contrast to absolute excess mortality rates, the estimates of cumulative pandemic RR for death during 1918–1921 were moderately associated with latitude; risk was highest in southern Chile (Spearman ρ = 0.48, p = 0.02; Figure 4) and provinces with low population density (Spearman ρ = −0.46, p = 0.03). Stepwise multivariate regression identified latitude, baseline mortality rates, and population density as predictors of cumulative RR for pandemic deaths across provinces (R^2^ = 51.5%, p = 0.003).

### Timing of Pandemic Waves and Patterns of Death by Age Group in Concepción

**Figure 5:**
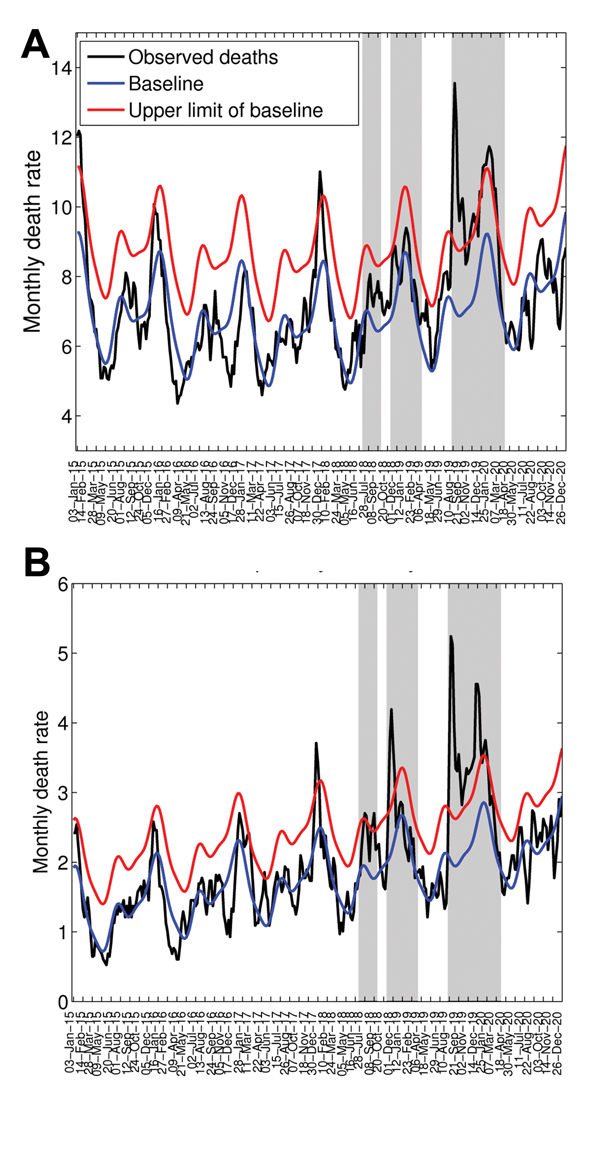
Weekly time series of deaths from all causes (A) and respiratory illness (B) per 10,000 population in Concepción, Chile, 1915–1920 (black lines). Shaded areas highlight 3 periods of high mortality rates associated with 3 waves of the pandemic occurring in July–September 1918, November 1918–March 1919, and August 1919–March 1920. Also shown are the Serfling seasonal regression model baseline (blue lines) and corresponding upper limit of the 95% confidence interval of the baseline (red lines).

**Figure 6:**
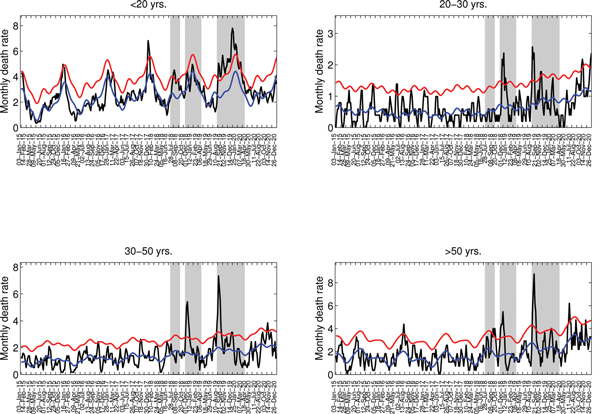
Age-stratified weekly respiratory mortality rates in Concepción, Chile, 1915–1920 (black lines). A) <20 years; B) 20–29 years; C) 30–50 years; D) >50 years. Shaded areas highlight 3 periods of excess deaths associated with 3 waves of the pandemic occurring in July–September 1918, November 1918–March 1919, and August 1919–March 1920. The Serfling seasonal regression model baseline (blue lines) and corresponding upper limit of the 95% confidence interval of the baseline (red lines) are also shown. Excess deaths are estimated as the number of deaths occurring above the upper limit of the baseline mortality curve, which was calibrated by using number of deaths before the 1918 influenza pandemic.

To explore timing of pandemic waves and patterns of death by age group in detail, we analyzed cause- and age-specific death rates for the city of Concepción during 1915–1920. Concepción experienced 3 periods of significantly elevated deaths from respiratory illness during 1918–1920: winter 1918 (July–September 1918), spring 1918–1919 (December 1918–January 1919), and winter–spring 1919–1920 (August 1919–February 1920). For these 3 putative pandemic waves, all-age rates of excess deaths from respiratory illness were estimated at 4, 7, and 34 deaths per 10,000 population, respectively (Table 2). Although the first local newspaper reports referring to the pandemic date back to October 24, 1918, the pandemic virus may have circulated at low levels 1–3 months earlier, July–September 1918, because all-age deaths from respiratory illness increased 31% above prepandemic baseline levels during this period (Figure 5, http://wwwnc.cdc.gov/EID/article/20/11/13-0632-F5.htm). The age patterns for this putative pandemic wave were mixed; significant excess deaths from respiratory illness occurred among children and persons >50 years of age (Figure 6), and a high number of all-cause deaths occurred among young adults 20–29 years of age.

**Table 2:** Estimated rates of absolute excess pandemic deaths, by pandemic wave, age group, and cause of death, Concepción, Chile, 1918–20*

Cause of death	Age group, no. excess deaths/10,000 population				
	<20 y	20–29 y	30–49 y	>50 y	All ages
Respiratory					
Jul 1918–Sep 1918	7.8	0.2	0.1	5.5	3.9
Nov 1918–Mar-1919	3.3	4.6	13.7	13.8	6.9
Aug 1919–Mar 1920	53.9	8.9	27.1	28	34.1
Total pandemic period	65.0	13.7	40.9	47.3	44.9
All-cause					
Jul 1918–Sep 1918	6.8	7.1	0.7	12.5	5.6
Nov 1918–Mar-1919	2.4	9.8	17.0	12.6	6.0
Aug 1919–Mar 1920	73.0	25.5	58.8	116.8	64.2
Total pandemic period	82.2	42.4	76.5	141.9	75.8

*Estimates of excess deaths were based on a seasonal regression model applied to weekly respiratory and all-cause mortality.

The occurrence of a subsequent pandemic wave in Concepción during December 1918–January 1919 is supported by newspaper reports. Although rates of absolute excess deaths were highest for older adults (Table 2) during this pandemic wave, the RR for death was highest among young adults 20–29 years of age, 54%–107% above baseline (Figure 6).

The onset of a third, severe and prolonged, pandemic wave in the city of Concepción was first noted in the local news on August 18, 1919. The August 1919–February 1920 pandemic wave in Concepción was synchronous with the major onslaught of the pandemic in other Chilean locales and was associated with the most deaths of the 3 pandemic waves. Excess death rates for respiratory illness and all causes were estimated at 34.1 and 64.2 deaths per 10,000 population, respectively. During this third wave, RR remained highest for young adults 20–29 years of age.

We cannot rule out the occurrence of a later pandemic wave in 1921 in Concepción because refined mortality data for this city were limited to 1915–1920. Overall, on the basis of respiratory and all-cause death data, we estimate that the cumulative effect of the pandemic during 1918–1920 in Concepción was 44.9–75.8 deaths per 10,000 population. Estimates for Concepción were similar, whether we aggregated city-level mortality data by week or month (within 90% of each other). Furthermore, our city-specific estimates were consistent with all-cause estimates obtained from the official vital statistics for the broader province.

## Discussion

Our analysis of extensive archival mortality statistics, death certificates, and contemporaneous newspaper reports reveals the substantial impact of the 1918–1921 pandemic in Chile. We found evidence that during 1918–1921, a total of 4 pandemic waves of varying timing and intensity occurred; in most areas of Chile, the highest rates of pandemic-related excess deaths were for July 1919–February 1920. Santiago was by far the hardest hit in terms of cumulative mortality burden. Pandemic-related excess mortality rates for 1918–1921 varied 10-fold across the 24 provinces; these differences were partly explained by latitude, baseline mortality rates, and population density. In agreement with previous reports (*4*–*7*,*9*–*12*), we found that among all age groups, the RR for pandemic death was highest among young adults 20–29 years of age. Our findings also indicate substantial excess mortality rates for senior populations, in agreement with previous reports from central Mexico (*6*) and Colombia (*7*).

We used monthly all-cause mortality data to estimate province-level mortality burden of the pandemic, as did prior studies (*30*–*32*). Because of the unusual severity of the 1918 pandemic, use of rather crude death indicators such as monthly all-cause deaths provides good agreement with more detailed cause-specific data (*4*,*26*,*30*,*31*). We cannot, however, rule out the possibility that underreporting (*1*) might have biased some of the geographic differences evidenced here. However, estimates of pandemic peak timing and excess deaths derived from refined death certificate data for the city of Concepción aligned with those derived from official all-cause mortality statistics for the broader province.

The temporal course of the pandemic in Chile differed markedly from that reported for the Northern Hemisphere. Although peak pandemic-related deaths occurred during October–November 1918 in temperate regions of the Northern Hemisphere (autumn) (*4**–**6**, **10**–**11*), the most lethal pandemic wave in Chile peaked in midwinter (August 1919). This pattern of delayed pandemic deaths in Chile echoes patterns reported from Argentina and Uruguay (*33*,*34*). Chile also experienced an earlier and milder wave of deaths during the warmer months (November 1918–February 1919), as did other South American localities including Peru (*9*), Colombia (*7*), Argentina (*33*), and Uruguay (*35*).

We suspect that a mild “herald” pandemic wave might have occurred in Concepción during July–September 1918, as did a concurrent mild pandemic wave in Lima, Peru (*9*). Indeed, we identified a significant elevation in total deaths among young adults 20–29 years of age during mid-1918 in Concepción but no increase in respiratory deaths in this signature age group. It is possible that the low and potentially unrecognized effect of this pandemic wave precluded identification of an increase in cause-specific deaths or that, alternatively, this elevation in deaths might be associated with a different pathogen. Herald waves occurring during March–August 1918 and associated with excess deaths concentrated among young adults have been reported for New York City (*4*), Mexico (*6*), Geneva (*36*), Copenhagen (*26*), the US military (*26*), the United Kingdom (*14*), and Singapore (*11*); these waves align with the laboratory identification of the pandemic influenza A(H1N1) virus from US soldiers in May 1918 (*37*). It is possible that other areas of Chile experienced mild pandemic waves during mid-1918 but that such waves could not be detected by using all-age, all-cause death data. In fact, the herald pandemic wave of 1918 in Denmark was only clearly evident from a sharp and unseasonal elevation in respiratory illness; there was little concurrent increase in all-cause or respiratory deaths (*26*). If the 1918 pandemic virus had indeed reached Chile by July 1918, only ≈4 months after the earliest putative outbreaks in New York City (*4*), this finding would indicate rapid transcontinental diffusion of the virus via boats and railways, reminiscent of the 1889 pandemic (*38*).

We found substantial variation in pandemic excess mortality rates across provinces of Chile. In particular, both relative and absolute risks for pandemic death during 1918–1921 were associated with baseline mortality rates. Furthermore, the highest relative mortality burden of the pandemic was found in southern Chile. Similarly, excess mortality rates associated with the 1918–1919 influenza pandemic were significantly higher in southern than in northern Europe (*30*). More broadly, substantial geographic heterogeneity in pandemic-associated mortality rates was reported globally and linked to socioeconomic factors (*15*). In addition to baseline death rates as predictors, we found that higher population density was predictive of higher absolute excess mortality rates.

Cumulative pandemic RR followed a north–south gradient for 1918–1921; this relative measure of pandemic death burden, which takes into account prepandemic baseline deaths, was highest in the southernmost provinces of Chile. We did not detect a southward gradient in total or infant mortality rates for the baseline prepandemic years, indicating that the pandemic-associated gradient was truly unique to this period and was not driven by reporting artifacts or socioeconomic conditions. Experimental studies indicate that influenza transmission is favored by lower temperatures and humidity levels (*39*), and we speculate that the higher relative death rate estimated for these areas could be explained by more favorable climate conditions in southern Chile (*18*,*40*). Moreover, the southward gradient in severity of the 1918 pandemic aligns with the patterns of the 2009 influenza A(H1N1) pandemic in Chile (*28*) and in Brazil (*23*). In contrast to patterns in RR for death, we did not find any association between latitude and cumulative absolute pandemic excess mortality rates during 1918–1921. Variations in absolute mortality rates seem to be mainly driven by underlying differences in baseline death rates and population density across Chile and hence do not support the climate hypothesis. Overall, the association between climate and influenza disease burden remains elusive, especially during pandemic seasons (*20*).

Excess mortality rates associated with the 1918 pandemic show substantial variability throughout Latin America, ranging from 0.4% in Boyacá, Colombia (*7*), to 2.9% in Iquitos, Peru (*9*). Our estimated cumulative mortality rate for Chile during 1918–1921 is 0.94%, which lies in the middle of the range of previous estimates for the broader region. At the province level, the highest pandemic excess mortality rate estimated was 2.1% for Santiago, which is comparable to that for Toluca, Mexico (*6*). In contrast, reports from the United States and Europe indicate lower mortality rates for these regions (0.5%–1.1%) (*4*,*5*,*30*).

Our detailed historical data from the city of Concepción, Chile, enabled us to analyze age-specific deaths and confirm the signature mortality risk among young adults (*4**–**7**,**10*,*12**,**26*). Furthermore, our data indicate that pandemic excess mortality rates were elevated among adults >50 years of age in Concepción, in agreement with data from Mexico (*6*) and Colombia (*7*), supporting a lack of senior sparing in Latin America, in contrast to the United States (*4*,*5*) and Europe (*10*,*26*). We and others have proposed that these age profile discrepancies originate from regional differences in prior immunity to the 1918 pandemic virus among seniors, resulting from the heterogeneous global circulation of influenza viruses in the 19th century (*6*). It is plausible that in the middle of the 19th century, remoteness could have affected the probability of introduction and dissemination of influenza viruses to Chile and the broader Latin American region.

In conclusion, our historical analysis reveals that the excess mortality rate of the 1918–1921 influenza pandemic was substantial in Chile, the southernmost region of Latin America, and that the major mortality burden was concentrated in the colder months of July 1919–February 1920, more than a year after the identification of the virus in the United States. Difference in pandemic excess mortality rates between Chilean provinces were >10-fold, and the gradient of excess deaths relative to baseline deaths increased from northern to southern Chile. Pandemic excess deaths were elevated for persons in all age groups, including seniors; these data are in agreement with data from other Latin American settings, although the signature atypical severity of the disease among young adults remained. Our findings suggest that a combination of local factors affected pandemic death patterns in Chile: variations in host-specific susceptibility, population density, baseline death rate, and climate conditions. We conclude that predicting death patterns for future pandemics is complex and multifactorial and that additional studies in undersampled areas are needed to refine our understanding of the predictors of mortality burden on a local scale.
